# Upregulated LINC01667 Expression Is Correlated With Poor Prognosis in Hepatocellular Carcinoma

**DOI:** 10.3389/fonc.2021.650173

**Published:** 2021-08-12

**Authors:** Kainan Zhang, Hui Liu, Mengsi Yu, Hui Zhao, Ning Yang, Xiaojuan Bi, Li Sun, Renyong Lin, Guodong Lü

**Affiliations:** ^1^ State Key Laboratory of Pathogenesis, Prevention, and Treatment of Central Asian High Incidence Diseases, Clinical Medical Research Institute, The First Affiliated Hospital of Xinjiang Medical University, Urumqi, China; ^2^ Graduate Academy, Xinjiang Medical University, Urumqi, China; ^3^ Department of Clinical Laboratory, The First Affiliated Hospital of Xinjiang Medical University, Urumqi, China; ^4^ College of Pharmacy, Xinjiang Medical University, Urumqi, China

**Keywords:** hepatocellular carcinoma, LINC01667, diagnostic biomarker, overall survival, carcinogenesis

## Abstract

The development of hepatocellular carcinoma (HCC) is a complex pathological process. Long intergenic non–protein-coding RNA 1667 (LINC01667, also known as MGC38584) plays an oncogenic role in several human cancers; however, its functional role in HCC tumorigenesis remains unknown. Here, we first evaluated the gene expression levels of LINC01667 in HCC using data from The Cancer Genome Atlas and Gene Expression Profiling Interactive Analysis (GEPIA) databases. We then elucidated the association between LINC01667 gene expression levels and the survival rates of patients with HCC. We detected the effect of LINC01667 on the malignant phenotypes (cell proliferation, migration, invasion and apoptosis etc.) and the MAPK and PI3K/AKT/mTOR signaling pathways of HepG2, SMMC-7721 and HUH7 cells. We also analyzed the sensitivity of HepG2, SMMC-7721 and HUH7 with different expression levels of LINC01667 to anti-HCC drugs *in vitro*. Based on data from the aforementioned databases and our experiments *in vitro*, we found that LINC01667 was overexpressed in HCC, and that patients with high LINC01667 levels had a remarkably poor overall survival rate. In addition, inhibition of LINC01667 expression suppressed the proliferation, migration and invasion of HepG2 and SMMC-7721 cells and promoted their apoptosis *in vitro*. In contrast, overexpression of LINC01667 promoted the proliferation, migration and invasion of HUH7 cells and suppressed their apoptosis *in vitro*. ChIRP-seq (chromatin isolation by RNA purification) showed that LINC01667 bound to MEG3, and downregulated the expression of MEG3. In addition, western blotting showed that LINC01667 could activate the NF-κB pathway to promote cancer progression. In conclusion, we report that LINC01667 is an important oncogene in HCC and may be used as a potential diagnostic and prognostic biomarker of HCC.

## Introduction

Hepatocellular carcinoma (HCC) is the second leading cause of cancer-related death worldwide, and has consequently become a major public health challenge (1–4). Its incidence is increasing at an alarming rate, which can be attributed to the high degree of resistance to chemotherapy and radiotherapy, lack of adequate vaccination against hepatitis B, and lack of consciousness and knowledge about the disease itself and a proper lifestyle (5). HCC is often associated with poor prognosis, and surgery is the only effective treatment method at present (6). No sensitive and specific biomarkers currently exist for HCC diagnostic and prognostic assessment (7); therefore, new studies need to focus on this research topic.

In recent years, the function and role of long intergenic non–protein-coding RNA (lncRNA) has been one of the hot spots in the field of tumor research. An increasing amount of evidence shows that lncRNAs play an important role in tumor development (8, 9). For example, many researchers have proven that MALAT1, a star lncRNA molecule in tumor research, is closely related to HCC progression. Hou et al. (10) found that MALAT1 promotes angiogenesis and the immunosuppressive properties of HCC cells by sponging miR-140.

LINC01667, also known as MGC38584, is a non-coding RNA that has been identified in recent years and is located on 21p11.2. The specific biological function of this gene is currently unknown, but related reports have noted that its expression may be related to the regulation of lung squamous cell carcinoma (11), but as of now, no research has reported that LINC01667 is related to HCC occurrence and development. Nevertheless, little remains known about the expression level and clinical significance of LINC01667 in HCC.

Herein, we aimed to explore the role of LINC01667 in HCC and evaluate its value as a prognostic marker for HCC. Using bioinformatics and *in vitro* functional experiments, we found that LINC01667 plays a critical role in HCC development and is an underestimated diagnostic and prognostic marker of HCC.

## Materials and Methods

### Data Collection

The gene expression profiles of LINC01667 were analyzed using The Cancer Genome Atlas (TCGA, https://www.cancer.gov/tcga) (12). We enrolled a small cohort of 20 patients with HCC at the First Affiliated Hospital of Xinjiang Medical University. All patients signed an informed consent form, and this study was reviewed by the Ethics Committee of the First Affiliated Hospital of Xinjiang Medical University.

### Vector Construction, Transfection and Stable Cell Line Construction

LINC01667 full-length sequences were generated by Genscript, Nanjing, China. Next, the LINC01667 sequences were cloned into the pHBLMV-CMV-MSC-EF1-fluc-T2A-puro (9831bp, Hanbio, shanghai, China) to generate the LINC01667 overexpression vector. To construct LINC01667-stably-overexpressed HCC cells, LINC01667 overexpression vector and control vector were transfected into HUH7 cells respectively. 48 hours after transfection, the cells were treated with 5 μg/ml neomycin for 4 weeks to select LINC01667-overexpressed HUH7 cells. All Smart Silencer(ssiLINC01667) and negative control were synthesized by RiboBio, Guangzhou, China(sequence):.

### Cell Culture and Antibodies

Human HCC cell lines (HepG2, Huh7, SMMC-7721, HCC-LM3, BEL-7402, Hep3B2.1-7) and normal liver L02 cells were obtained from American Type Culture Collection (Manassas, VA, USA). The cells were cultured in Dulbecco’s modified Eagle’s medium (DMEM, Gibco BRL, Grand Island, NY, USA) supplemented with 10% fetal bovine serum, 100 U/mL penicillin, and 100 μg/mL streptomycin in a humidified atmosphere of 5% CO_2_.

### Quantitative RT-PCR

Total RNA was extracted using TRIzol (St. Louis, MO, USA). RNA purity and concentrations were determined using a NanoDrop 2000 spectrophotometer (Thermo Fisher Scientific, Waltham, MA, USA). First, we used complementary DNA (cDNA) synthesis kits (HiScript Reverse Transcriptase, Vazyme, Nanjing, China) according to the kit protocols. SYBR Green RT-PCR was performed to measure lncRNA levels, which were then calculated using the comparative threshold cycle (2-ΔΔCt) method. The LINC01667 forward and reverse primers were as follows: 5′-GATGACAGCAGTCGCAAAG-3′ and 5′-GACAGTGACCCAACCAACA-3′, respectively.

### Cell Proliferation, Migration, Invasion, Apoptosis, and Cell Cycle Analysis

Cell viability was measured using the EdU (5-ethynyl-2′-deoxyuridine) assay. Cell migration was evaluated using the Transwell assay. Cell invasion was assessed using the BioCoat Matrigel Invasion Chamber (BD Biosciences). The cell numbers for cell migration and invasion were counted in three random fields. The cells were stained with annexin V and propidium iodide (PI) using an Annexin V–FITC Apoptosis Detection kit (Invitrogen), and the percentage of apoptotic cells was examined with flow cytometry (Beckman Coulter, Brea, CA, USA). For detecting the cell cycle, the cells were stained with PI after 48-h transfection, and examined by fluorescence-activated cell sorting (FACS). All experiments were repeated three times (13, 14).

### Chromatin Isolation by RNA Purification

HepG2 cells (2 × 10^6^ cells per assay) were cross-linked with 1% formaldehyde at room temperature for 10 min, and quenched in 125 mM glycine for 5 min. The precipitated DNA was purified using a ChIRP-Kit (BersinBio, Guangzhou, China). The HepG2 cells were cross-linked, lysed and sonicated, and incubated with odd or even probe sets, and LINC01667-bound chromatin was retrieved. The subsequent sequencing work was completed by RiboBio (Guangzhou, China).

### Western Blotting

The cells were lysed in 1% Triton X-100 lysis buffer. Total protein concentrations in the cell lysates were determined using a bicinchoninic acid (BCA) protein assay kit (Pierce, Rockford, IL, USA). The proteins from each sample were separated by 10% sodium dodecyl sulfate–polyacrylamide gel electrophoresis (SDS-PAGE) and transferred to a polyvinylidene fluoride membrane. The membranes were blocked with Tris-buffered saline (TBS) containing 5% non-fat milk powder at 37°C for 2 h, and immunoblot analysis was performed with antibodies at 4°C for 12 h. The membranes were washed with TBS–Tween 20 (TBS-T) buffer three times, followed by incubation with horseradish peroxidase (HRP)-conjugated polyclonal secondary antibody for 1 h at 37°C. The membranes were developed using the enhanced plus chemiluminescence assay (Pierce) according to the manufacturer’s instructions. Finally, images were analyzed using Image-Pro Plus 6.0.

### *In Vitro* Sorafenib/Regorafenib Exposure

*In vitro* Sorafenib/Regorafenib exposure experiments were carried out as described by literature reports (15–17). Briefly, Sorafenib/Regorafenib (20 μM/5 μM) was added. Cell growth was assayed for up to 72 h. CCK-8 was used to detect cell proliferation at 0, 12, 24, 48 and 72 hours, respectively. All experiments were repeated five times.

### Statistical Analysis

All statistical analyses were performed using SPSS 22.0 software (IBM SPSS, Chicago, IL, USA) and R 3.6.1 (R Foundation for Statistical Computing, Vienna, Austria) (18). The correlation of LINC01667 expression with clinical characteristics was detected using χ^2^ tests. Overall survival (OS) was analyzed by Kaplan–Meier survival curves, and the differences between subgroups were compared by log-rank tests. *P* < 0.05 was considered statistically significant.

## Results

### LINC01667 Expression Is Significantly Upregulated in HCC

**Figure 1:**
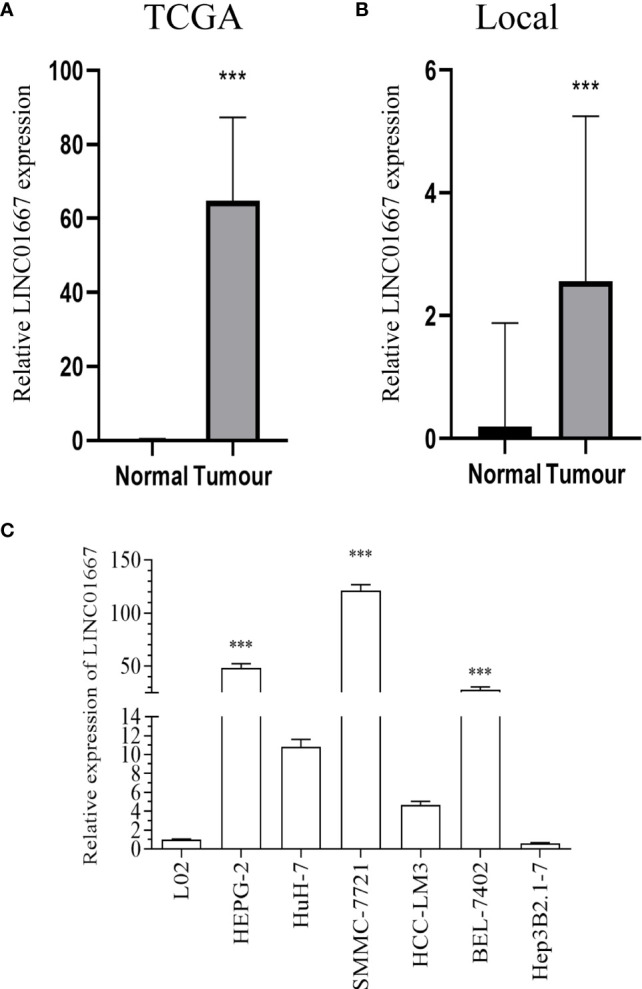
LINC01667 was upregulated in HCC. **(A)** Relative LINC01667 expression in HCC tissues from patients *versus* normal samples from TCGA database (normal = 50, tumor = 374). **(B)** Relative LINC01667 expression in HCC tissues from patients *versus* normal samples from the local cohort (normal = 20, tumor = 20). ***P < 0.05. **(C)** In comparison with L02 cells, LINC01667 was upregulated in HCC cells (HepG2, Huh7, SMMC-7721, HCC-LM3, BEL-7402, Hep3B2.1-7). ***P < 0.001.

To explore the potential role of LINC01667 in HCC, LINC01667 expression was analyzed using TCGA database. LINC01667 expression was significantly higher in HCC cases (n = 374) than in the healthy controls (n = 50) (*P* < 0.001) (**Figure 1A**). To clarify LINC01667 expression in HCC, the cancer and para-cancer specimens of 20 patients with HCC were used for detection. In the 20 pairs of tissue samples we collected, LINC01667 expression in the cancer tissues was also higher than that in normal tissues (**Figure 1B**). To verify LINC01667 expression in HCC, we detected its expression in HCC cell lines and normal cell lines. Consistent with the findings from the HCC tissues, the qRT-PCR showed that LINC01667 was overexpressed in HCC cell lines (HepG2, SMMC-7721, BEL-7402) compared with the normal liver L02 cell line (*P* < 0.001) (**Figure 1C**).

### LINC01667 Has High Diagnostic Value for HCC

**Figure 2:**
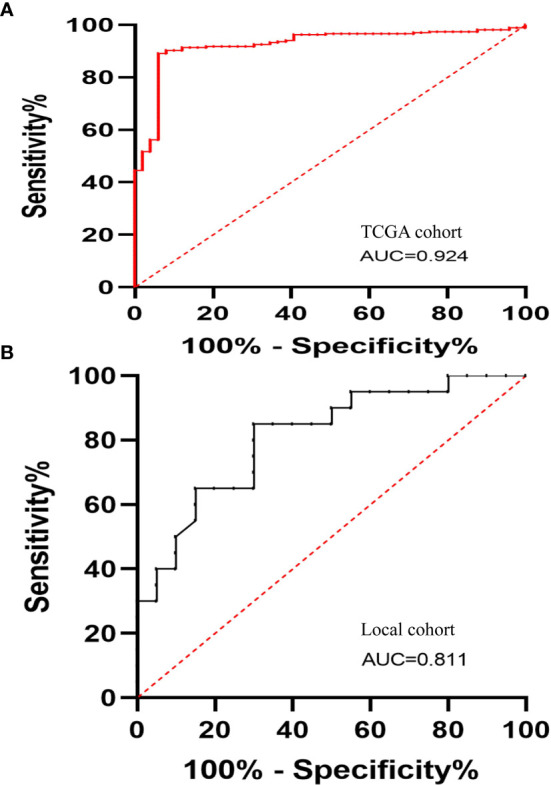
The diagnostic ROC curves of LINC01667 in HCC. **(A)** Diagnostic ROC curves of LINC01667 for distinguishing patients with HCC and normal controls in a TCGA cohort (normal = 49, tumor = 267). **(B)** Diagnostic ROC curves of LINC01667 for distinguishing patients with HCC and normal controls in a local cohort (normal = 20, tumor = 20).

We explored the possible potential application of LINC01667 for distinguishing HCC. The diagnostic receiver operating characteristic (ROC) analysis of LINC01667 in TCGA cohort showed that LINC01667 can serve as a potential diagnostic biomarker for HCC (*P* < 0.001). **Figure 2A** shows that LINC01667 [area under the ROC curve [AUC] (95% confidence interval [CI]) = 0.924 (0.887–0.962)] exhibited high diagnostic value, with 89.14% sensitivity and 93.88% specificity (*P* < 0.001). At the same time, our local small cohort yielded similar results. **Figure 2B** shows that LINC01667 [AUC (95% CI) = 0.811 (0.679–0.944)] exhibited high diagnostic value, with 65% sensitivity and 85% specificity (*P* < 0.001).

### High LINC01667 Expression Predicts Poor Prognosis for Patients With HCC

**Figure 3:**
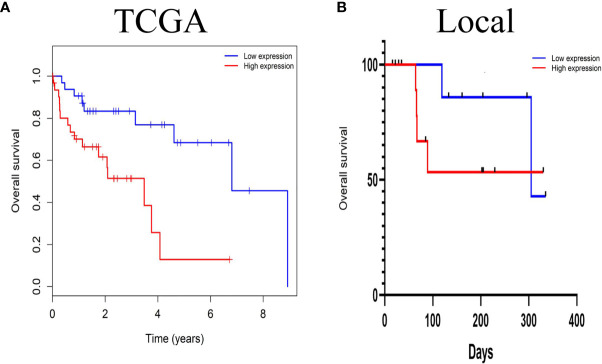
The prognostic significance of LINC01667 in patients with HCC. **(A)** Kaplan–Meier curves showing the OS of patients with HCC according to high and low LINC01667 expression in a TCGA cohort (n = 374). **(B)** Kaplan–Meier curves showing the OS of patients with HCC according to high and low LINC01667 expression in the local cohort (n = 20).

The prognostic effects of LINC01667 for the OS of patients with HCC were evaluated by Kaplan–Meier survival analysis and the log-rank test. Patients with upregulated LINC01667 expression had shorter OS than the patients with low LINC01667 expression (*P*<0.001) (**Figure 3A**). Of course, to test whether situations are similar for different regions and races, we performed the same survival analysis on the local 20-patient cohort. Although the graph shows that the patients with low LINC01667 expression had higher survival probability than patients in the high-expression group, unfortunately, there was no statistical difference (*P*=0.2825), but we believe this is due to the small sample size (**Figure 3B**). To explore the role of LINC01667 in HCC, we performed a preliminary analysis to identify whether LINC01667 expression in HCC tissue from patients is associated with clinicopathological parameters. Based on the median value of LINC01667 expression, the patients were divided into high and low LINC01667 expression groups. χ^2^ tests revealed the relationship between LINC01667 expression and the patients’ clinical characteristics. **Table 1** shows that high LINC01667 expression in patients with HCC is significantly related to age (*P* < 0.001).

**Table 1:** Correlations between LINC01667 expression and clinicopathological characteristics in HCC.

Characteristics		Total	LINC01667		*P*
			High	Low	
Age	≥49	213	118	95	<0.001
	<49	52	15	37	
	NA	1			
Gender	male	181	89	92	0.7
	female	85	44	41	
Stage	I	125	58	67	0.672
	II	62	32	30	
	III+IV	61	32	29	
	NA	18			
T	T1+T2	201	99	102	0.787
	T3+T4	63	34	29	
	NA	2			
Space	Asian	119	51	68	0.158
	black or African American	16	10	6	
	white	123	65	58	
	NA	8			

### LINC01667 Knockdown Inhibits Cell Proliferation, Migration, and Invasion, and Promotes Apoptosis of HepG2 and SMMC-7721 *In Vitro*

**Figure 4:**
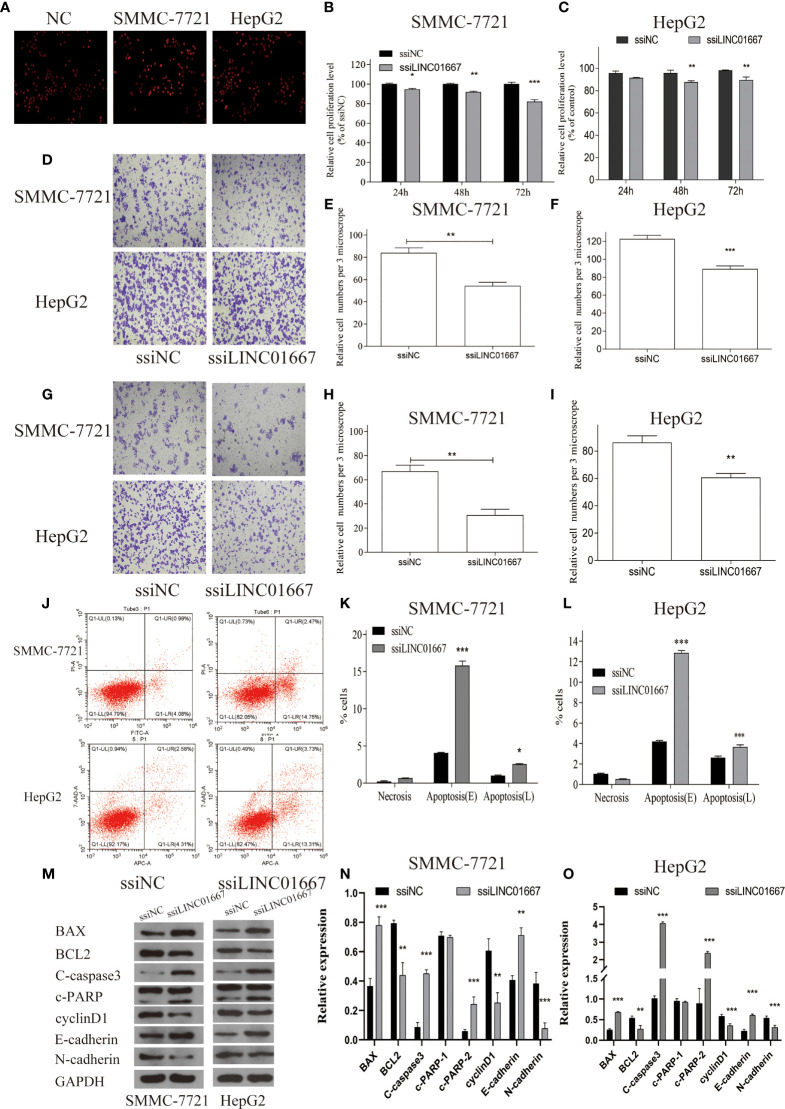
Knockdown of LINC01667 inhibited cell proliferation, migration, and invasion, and promoted apoptosis *in vitro*. **(A)** EDU staining of different groups at 48h. **(B, C)** Proliferation potential in SMMC-7721 and HepG2 cell lines that inhibited of LINC01667 at 24, 48 and 72h. **P < 0.01, ***P< 0.001. **(D)** The transwell experiment was used to detect the migration potential of cells. **(E, F)** Migration potential in SMMC-7721 and HepG2 cell lines hat inhibited of LINC01667at 48h. **P < 0.01, ***P < 0.001. **(G)** The transwell experiment was used to detect the Invasion potential of cells. **(H, I)** Invasion potential in SMMC-7721 and HepG2 cell lines at 48 h in LINC01667 knock downing cells. **P < 0.01, ***P< 0.001. **(J)** Flow cytometry experiments was used to detect the apoptosis potential. **(K, L)** LINC01667 knockdown increased cell apoptosis (***P < 0.001). **(M–O)** Western blot was used to analysis the expression of BCL-2, cyclin D1, N-cadherin, Bax, C-caspase3, c-PARP2 and E-cadherin in HepG2/SMMC-7721 cells. *P < 0.05. **P < 0.01, ***P < 0.001. ssiNC represents the random sequence group, and ssiLINC01667 represents the knockdown LINC01667 group.

Considering that LINC01667 was upregulated in HCC tissues, we next investigated the effects of LINC01667 silencing on HCC cell. Since HepG2 and SMMC-7721 cells have relatively high expression level of LINC01667, these two cells were selected for the experiment, as verified by qRT-PCR (**Supplementary Figure 1**). Intriguingly, silencing LINC01667 significantly inhibited HepG2 and SMMC-7721 cell proliferation potential (**Figures 4A–C**); moreover, the migration and invasion abilities of the cells were significantly suppressed (**Figures 4D–I**). Flow cytometry demonstrated that, compared with the ssiNC, ssiLINC01667 significantly increased the apoptosis of HepG2 and SMMC-7721 cells (**Figures 4J–L**). Western blotting showed that LINC01667 knockdown caused the downregulation of BCL-2, cyclin D1 and N-cadherin expression, whereas Bax, C-caspase3, c-PARP2 and E-cadherin expression was upregulated(**Figures 4M–O**). In addition, key proteins in MAPK and PI3K/AKT/mTOR signaling pathways have also been tested, as verified by WB (**Supplementary Figure 2**), prompt that LINC01667 could activate the above-mentioned pathway.

### LINC01667 Overexpression Promoted Cell Proliferation, Migration and Invasion, and Inhibited Cell Apoptosis of HUH7 *In Vitro*

**Figure 5:**
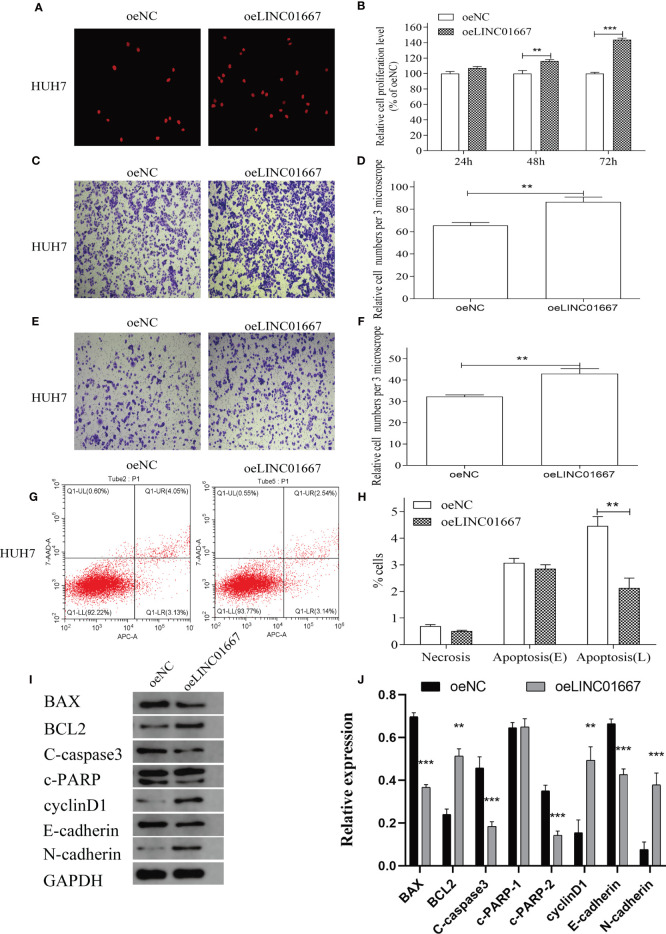
Overexpression of LINC01667 in HUH7 cells can promote cell proliferation, migration, and invasion, and inhibit apoptosis *in vitro*. **(A, B)** Proliferation potential in HUH7 cell lines at 24, 48 and 72h in stable LINC01667 overexpressing cells. **P < 0.01, ***P < 0.001. **(C, D)** Migration potential in HUH7 cell lines at 48h in stable LINC01667 overexpressing cells. **P < 0.01, ***P < 0.001. **(E, F)** Invasion potential in HUH7 cell lines at 48h in stable LINC01667 overexpressing cells. **P < 0.01, ***P < 0.001. **(G, H)** Overexpressed of LINC01667 inhibited cell apoptosis (***P < 0.001). **(I, J)** Western blot was used to analysis the expression of BCL-2, cyclin D1, N-cadherin, Bax, C-caspase3, c-PARP2 and E-cadherin in HUH7 cells. **P < 0.01, ***P < 0.001. oeNC represents the empty vector group, and oeLINC01667 represents the overexpression LINC01667 group.

In order to further verify the relationship between the expression level of LINC01667 and malignant phenotype of HCC cells, we overexpressed LINC01667 in the HUH7 cell line with a relatively low expression level of LINC01667, as verified by qRT-PCR (**Supplementary Figure 1**). The results showed that overexpression of LINC01667 promoted the cell proliferation, invasion and migration of HUH7 cell line (**Figures 5A–F**), and at the same time, the cell apoptosis of HUH7 cell line was inhibited (**Figures 5G, H**). Western blotting showed that LINC01667 overexpression upregulated the expression of BCL-2, cyclin D1, and N-cadherin in HUH7 cell line, whereas the expression of Bax, C-caspase3, c-PARP2 and E-cadherin in HUH7 cell line was downregulated (**Figures 5I, J**). In addition, key proteins in MAPK and PI3K/AKT/mTOR signaling pathways have also been tested, as verified by WB (**Supplementary Figure 2**), prompt that LINC01667 could activate the above-mentioned pathway.

### LINC01667 Activates the NF-κB Pathway by Downregulating MEG3 Expression

**Figure 6:**
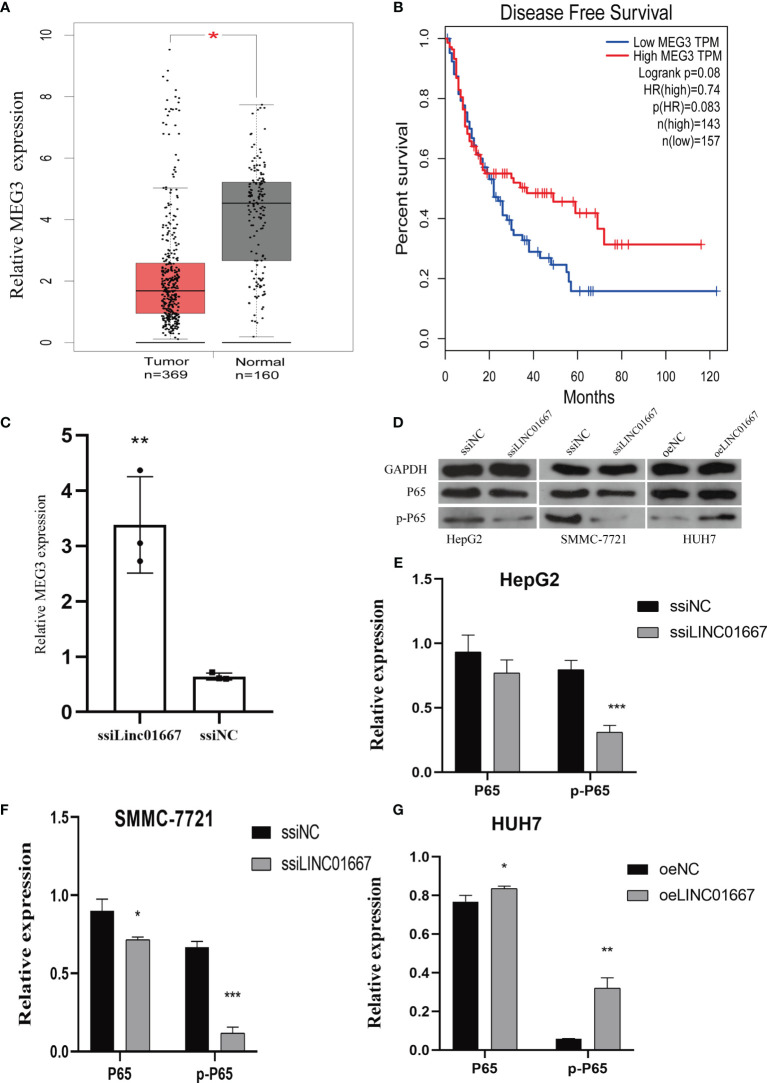
LINC01667 has a regulatory relationship with MEG3 and NF-κB. **(A)** *MEG3* expression levels in TCGA and GEPIA cohorts (normal = 160, tumor = 369, *P  < 0.05). **(B)** Kaplan–Meier curves showing the OS of patients with HCC according to high and low LINC01667 expression in a TCGA cohort (n = 300). **(C)** Knockdown of LINC01667 increases the expression of MEG3. **(D–G)** LINC01667 could activate the NF-κB pathway. *P < 0.05. **P < 0.01, ***P < 0.001. oeNC represents the empty vector group, and oeLINC01667 represents the overexpression LINC01667 group, ssiNC represents the random sequence, and ssiLINC01667 represents the knockdown LINC01667 group.

ChIRP-Seq showed that LINC01667 binds to MEG3. In addition, we found in the GEPIA database that MEG3 is downregulated in tumor tissues, which is the opposite of the expression trend of LINC01667 (**Figure 6A**). Moreover, we found that patients with high MEG3 expression had longer OS (**Figure 6B**). Furthermore, we observed upregulated MEG3 expression after knocking down LINC01667, indicating that LINC01667 can bind MEG3 and cause its down-regulation (**Figure 6C**). The western blotting showed that knockdown inhibited the phosphorylation of P65, while overexpression of LINC01667 promoted the activation of P65, which indicated that LINC01667 could promote the development of cancer by activating the NF-κB pathway (**Figures 6D–G**).

### LINC01667 May Increase the Chemical Sensitivity of Sorafenib and Regorafenib, but No Synergistic Effect

**Figure 7:**
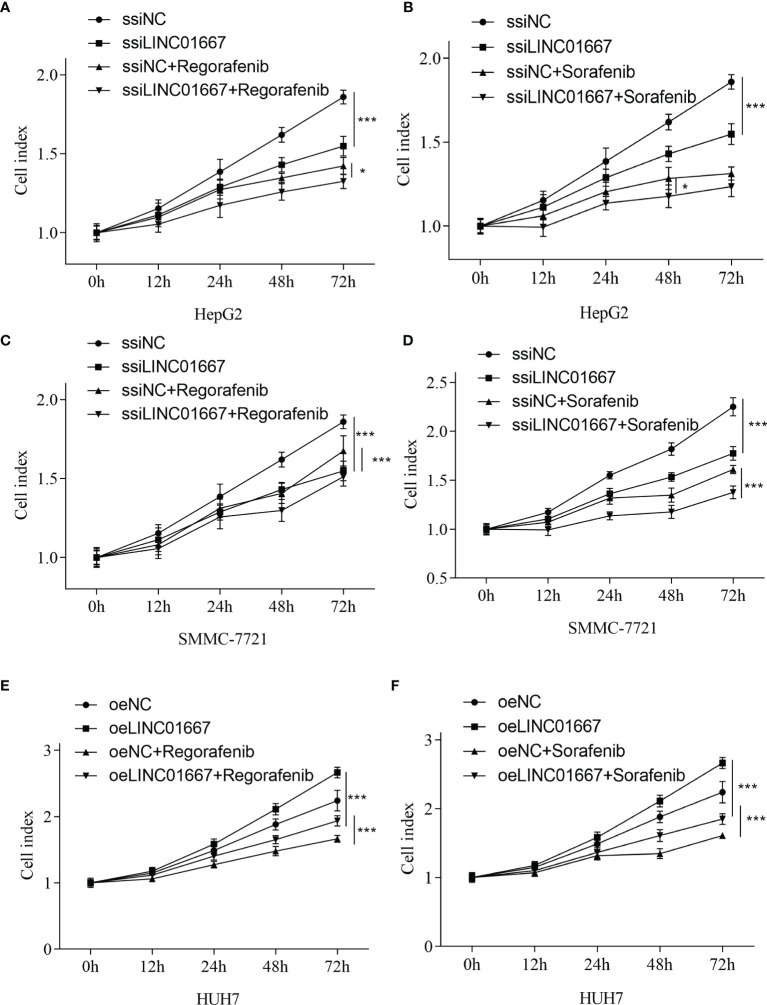
LINC01667 modulates Sorafenib and Regorafenib response in HCC cells. Effect of Regorafenib treatment in cells with **(A, C)** knockdown of LINC01667 (HepG2 and SMMC-7721) or **(E)** stable overexpression of LINC01667 (HUH7) compared with the transfected controls. Effect of Sorafenib treatment in cells with **(B, D)** knockdown of LINC01667 (HepG2 and SMMC-7721) or **(F)** stable overexpression of LINC01667 (HUH7) compared with the transfected controls. All experiments were performed in 5 copies. *P < 0.05, ***P < 0.001. oeNC represents the empty vector group, and oeLINC01667 represents the overexpression LINC01667 group, ssiNC represents the random sequence group, and ssiLINC01667 represents the knockdown LINC01667 group.

A large number of studies have pointed out that the expression of lncRNA is related to the resistance of anti-tumor drugs (19–21). We sought to investigate the impact of LINC01667 modulation in HCC-derived cell lines upon Sorafenib and Regorafenib exposure *in vitro*. In addition to the HCC-derived cell lines (HUH7) with stable LINC01667 overexpression, we also generated two cell lines (SMMC-7721 and HepG2) with LINC01667 knockdown. The results showed that LINC01667 could promote the proliferation of HCC cell lines. When LINC01667 was knocked down, it could inhibit cell proliferation, while overexpression of LINC01667 could promote cell proliferation. In addition, when Sorafenib and Regorafenib were added, the proliferation rate of cells was decreased. Knocked down LINC01667 and adding anti-tumor drugs would more significantly inhibit the proliferation rate of cells (**Figure 7**). Furthermore, the Bliss independence criterion showed that there was no synergistic effect between LINCO1667 inhibition and Sorafenib/Regorafenib.

## Discussion

HCC is difficult to diagnose in its early stages. Therefore, there is an urgent need to identify sensitive and specific biomarkers for improving HCC diagnosis and for accurately evaluating its prognosis. LncRNAs can promote the development of HCC and can be used as diagnostic biomarkers (8, 9, 22), such as the lncRNA HEIH in serum and exosomes as a potential biomarker in the HCC (23), but it is necessary to discover new, potentially better lncRNAs as indicators. Herein, we report that LINC01667 plays a critical role in HCC occurrence and development and has great value as a diagnostic and prognostic biomarker of HCC.

LINC01667 appears to be an effective diagnostic/prognostic biomarker for HCC. On one hand, according to TCGA, we found that LINC01667 had an AUC of >0.92, and based on the local cohort, the AUC was >0.81, with the value being superior to that of alpha-fetoprotein (AFP, 41%–65% sensitivity and 80%–94% specificity for elevated serum AFP levels of >20 ng/mL in diagnosing HCC) (24), one of the most common diagnostic biomarkers for HCC. Moreover, we found that upregulated expression levels of LINC01667 were associated with shorter OS in patients with HCC, implying that LINC01667 can serve as a biomarker for predicting HCC progression. According to Kawai et al., who reported that SOX9 is a novel HCC/cancer stem cell marker, patients with SOX9^+^ tumors exhibited significantly poorer recurrence-free survival, but no significant difference was observed for OS (25). Therefore, LINC01667 may be used as an independent indicator, and has greater evaluation efficiency than that using SOX9 alone. On the other hand, LINC01667 expression levels are a good predictor of the survival probability of patients. Our results suggest that whether it is TCGA database or small local cohort verification, patients with high LINC01667 expression have shorter survival times. This shows that detecting LINC01667 can help clinicians assess the severity of the patient’s condition effectively. However, we have not found that LINC01667 expression level is related to the clinical stage of HCC. There may be two reasons for this phenomenon: 1) The expression level of LINC01667 does not increase gradually with disease development; 2) LINC01667 expression peaks in the early stage of disease. The present results cannot fully determine which of the above reasons is correct. However, if LINC01667 expression levels are higher in the early stage of the disease, higher sensitivity will be obtained during clinical use.

LINC01667 is involved in HCC occurrence and development. LINC01667 can act as a sponge to regulate genes such as CCNB1, CEP55, MKI67, and TYMS, and ultimately affect the progression of lung adenocarcinoma (26). However, there is no report on the specific biological function of LINC01667. As our results show, knockdown of LINC01667 inhibited cell proliferation, migration, and invasion, and promoted apoptosis *in vitro*, overexpression of LINC01667 will also get similar results, suggesting that high expression levels of LINC01667 may lead to poor prognoses. Moreover, western blotting has shown that LINC01667 may mediate epithelial–mesenchymal transition (EMT) (27). This may be an important factor in LINC01667 promotion of HCC progression. There may be two main reasons LINC01667 can promote HCC progression. One is that the expression of MEG3 will also be upregulated when LINC01667 expression is knocked down, which means that LINC01667 may regulate MEG3 expression at the transcriptional level. The biological function of MEG3 in repressing tumor through regulating the major tumor suppressor genes p53 and Rb, inhibiting angiogenesis-related factor, or controlling miRNAs has been highlighted (28). On the other hand, previous studies have also suggested that MEG3 mediates EMT (29, 30). This is consistent with our present *in vitro* results. Another point is the function of the NF-κB pathway (31). Our results show that P-P65 levels increased when LINC01667 was knocked down, which indicates that LINC01667 may have the effect of activating the NF-κB pathway, thereby promoting the malignant progression of tumors. However, because LINC01667 may cause changes in MEG3 expression levels, and MEG3 can also activate the NF-κB pathway (32–34), we are currently unable to determine whether there is a synergistic effect between LINC01667 and MEG3, and the relevant regulatory mechanism requires further study. In addition to the possible mechanisms mentioned above, LINC01667 may also promote MAPK and PI3K/AKT/mTOR signaling pathways, but how LINC01667 activates these pathways still needs further research.

Furthermore, considering the clinical treatment plan for HCC patients, we conducted *in vitro* drug exposure experiments on HCC cell lines with different expression levels of LINC01667. The results found that LINC01667 can promote the resistance of HCC cell lines to anti-tumor drugs. This suggested that LINC01667 can be considered as a target for anti-HCC drugs.

In summary, our study proves for the first time that LINC01667 expression is increased significantly in HCC, and that the high LINC01667 expression can lead to the malignant progression of HCC; second, LINC01667 can be used as an effective biomarker for diagnosing HCC and evaluating survival time. Further, it has potential treatment targets for follow-up research.

## Data Availability Statement

The raw data supporting the conclusions of this article will be made available by the authors, without undue reservation, to any qualified researcher.

## Author Contributions

KZ and HL conceived the project and edited the manuscript. MY carried out the experiments in this study. HZ, NY, XB, and LS contributed to manuscript preparation. HZ contributed to the collection of all clinical samples and clinical backgrounds. RL and GL were the principal investigators who designed and conceived the study and obtained financial support. All authors contributed to the article and approved the submitted version.

## Funding

This study was funded by the Natural Science Foundation of Xinjiang Uygur Autonomous Region, PR China (2018D01C199).

## Conflict of Interest

The authors declare that the research was conducted in the absence of any commercial or financial relationships that could be construed as a potential conflict of interest.

## Publisher’s Note

All claims expressed in this article are solely those of the authors and do not necessarily represent those of their affiliated organizations, or those of the publisher, the editors and the reviewers. Any product that may be evaluated in this article, or claim that may be made by its manufacturer, is not guaranteed or endorsed by the publisher.
