# Corrigendum: Upregulated LINC01667 Expression Is Correlated With Poor Prognosis in Hepatocellular Carcinoma

**DOI:** 10.3389/fonc.2021.785394

**Published:** 2021-10-15

**Authors:** Kainan Zhang, Hui Liu, Mengsi Yu, Hui Zhao, Ning Yang, Xiaojuan Bi, Li Sun, Renyong Lin, Guodong Lü

**Affiliations:** ^1^ State Key Laboratory of Pathogenesis, Prevention, and Treatment of Central Asian High Incidence Diseases, Clinical Medical Research Institute, The First Affiliated Hospital of Xinjiang Medical University, Urumqi, China; ^2^ Graduate Academy, Xinjiang Medical University, Urumqi, China; ^3^ Department of Clinical Laboratory, The First Affiliated Hospital of Xinjiang Medical University, Urumqi, China; ^4^ College of Pharmacy, Xinjiang Medical University, Urumqi, China

**Keywords:** hepatocellular carcinoma, LINC01667, diagnostic biomarker, overall survival, carcinogenesis

In the original article, there was a mistake in ***Figure 6G*** as published. **The label of Figure 6G should be HUH7 instead of HUH7-p65.** The corrected ***Figure 6*** appears below.

In the original article, there was a mistake in ***Figure 7 E, F*** as published. **During the last round of revisions, we uploaded the wrong version of the file, resulting in a complete repetition of images E, F and C, D. “E, F” should have been the result of the HUH7 cell line.** The corrected ***Figure 7*** appears below.

**Figure 6:**
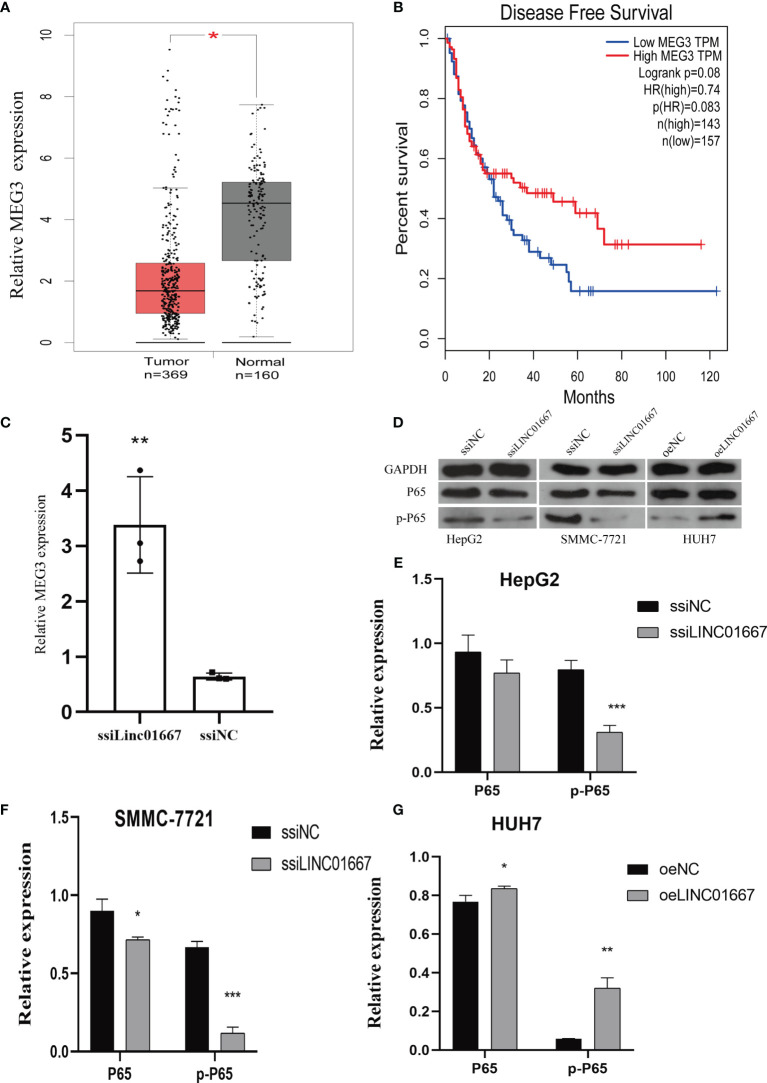
LINC01667 has a regulatory relationship with MEG3 and NF-kB. **(A)** MEG3 expression levels in TCGA and GEPIA cohorts (normal = 160, tumor = 369, *P < 0.05). **(B)** Kaplan–Meier curves showing the DFS of patients with HCC according to high and low MEG3 expression in a TCGA cohort (n = 300). **(C)** Knockdown of LINC01667 increases the expression of MEG3. **(D–G)** LINC01667 could activate the NF-kB pathway. *P < 0.05. **P < 0.01, ***P < 0.001. oeNC represents the empty vector group, and oeLINC01667 represents the overexpression LINC01667 group, ssiNC represents the random sequence, and ssiLINC01667 represents the knockdown LINC01667 group.

**Figure 7:**
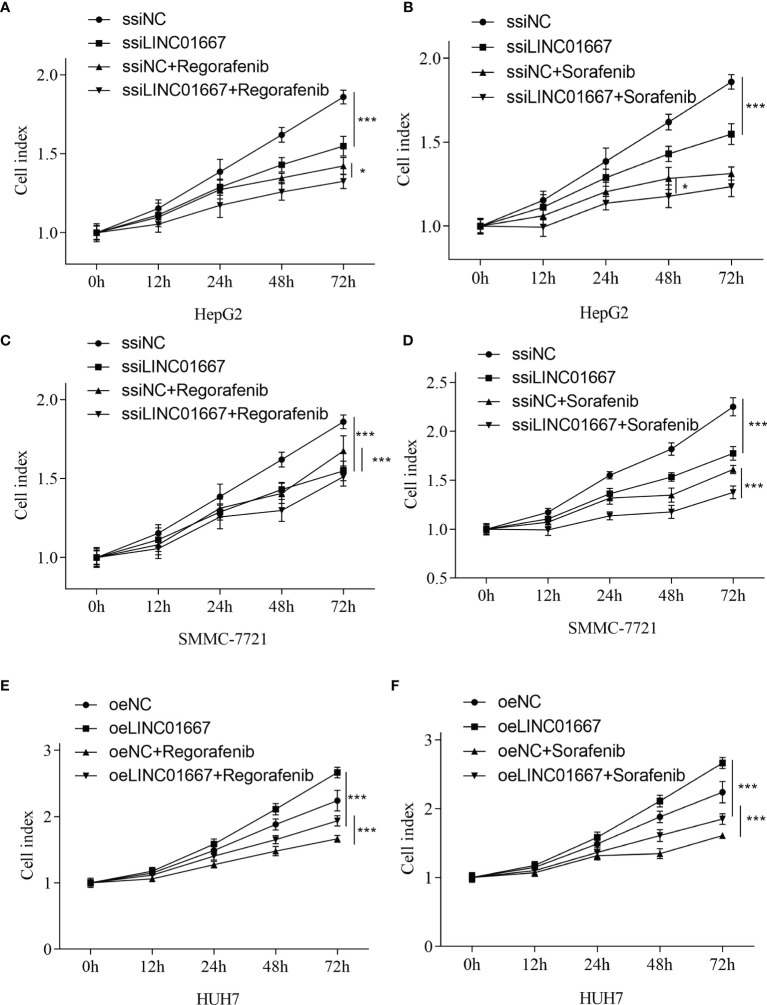
LINC01667 modulates Sorafenib and Regorafenib response in HCC cells. Effect of Regorafenib treatment in cells with **(A, C)** knockdown of LINC01667 (HepG2 and SMMC-7721) or **(E)** stable overexpression of LINC01667 (HUH7) compared with the transfected controls. Effect of Sorafenib treatment in cells with **(B, D)** knockdown of LINC01667 (HepG2 and SMMC-7721) or **(F)** stable overexpression of LINC01667 (HUH7) compared with the transfected controls. All experiments were performed in 5 copies. *P < 0.05, ***P < 0.001. oeNC represents the empty vector group, and oeLINC01667 represents the overexpression LINC01667 group, ssiNC represents the random sequence group, and ssiLINC01667 represents the knockdown LINC01667 group.

The authors apologize for these errors and state that this does not change the scientific conclusions of the article in any way. The original article has been updated.

## Publisher’s Note

All claims expressed in this article are solely those of the authors and do not necessarily represent those of their affiliated organizations, or those of the publisher, the editors and the reviewers. Any product that may be evaluated in this article, or claim that may be made by its manufacturer, is not guaranteed or endorsed by the publisher.

