# Supplementary figures and images for: Upregulated LINC01667 Expression Is Correlated With Poor Prognosis in Hepatocellular Carcinoma
Source: Front Oncol. 2021 Aug 12;11:650173. doi: 10.3389/fonc.2021.650173 (PMC8397520; doi:10.3389/fonc.2021.650173)

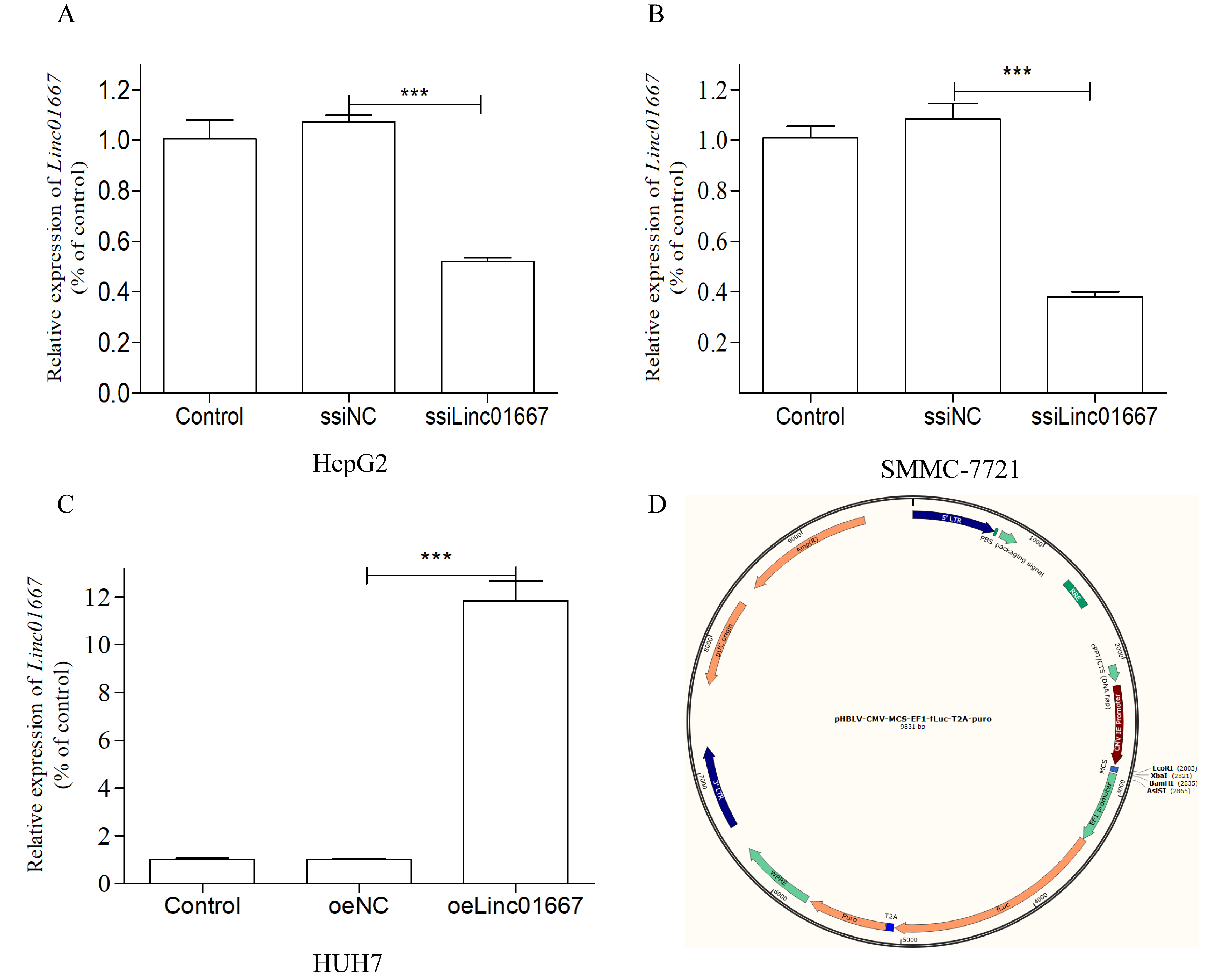

Supplement: Supplementary file 1 [file Image_1.tif]

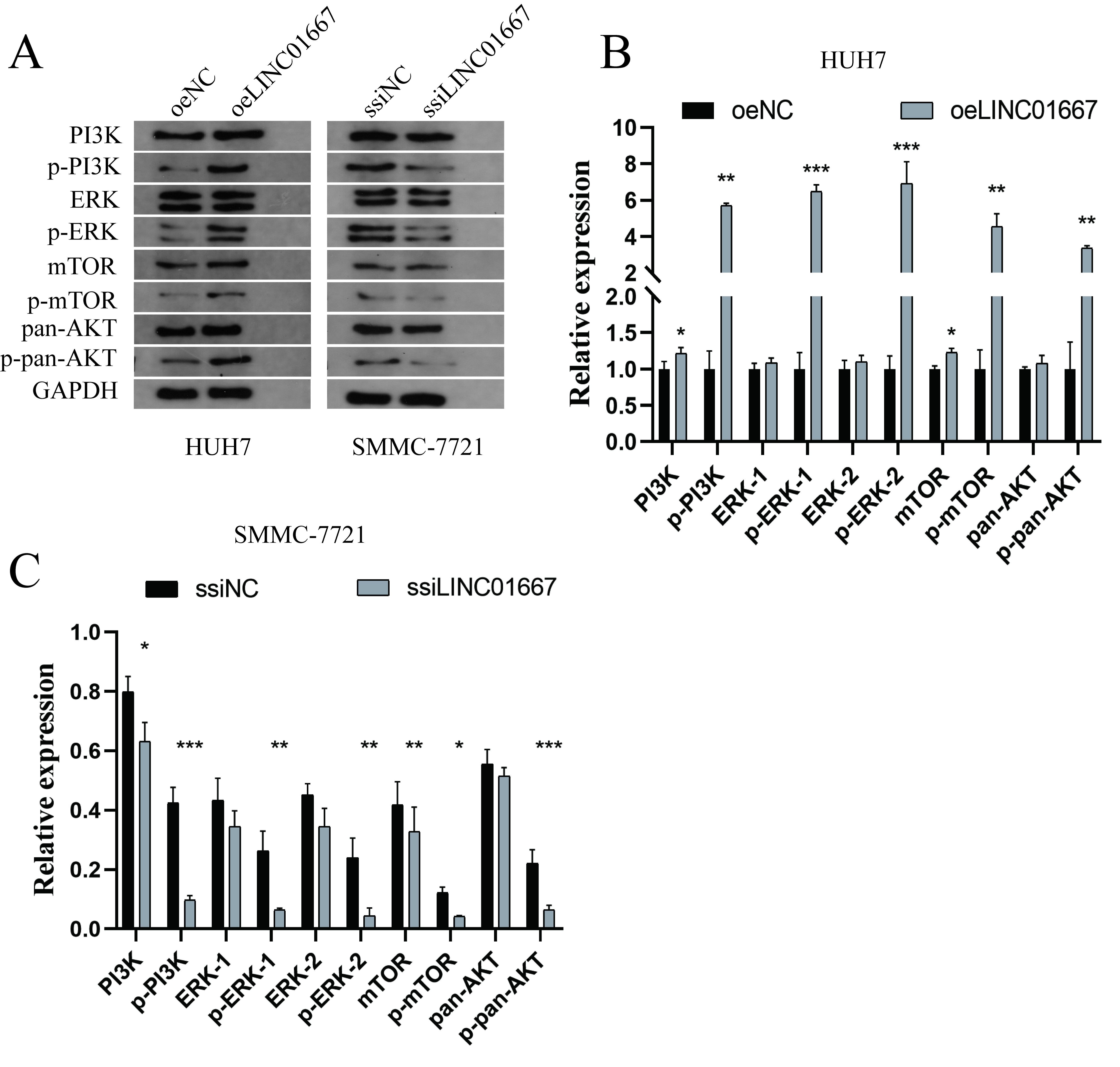

Supplement: Supplementary file 2 [file Image_2.tif]
